# Case of Fungus Hæmatodes of the Brain, with Long-Continued Constipation of the Bowels

**Published:** 1831-11

**Authors:** Jas. Laidlaw

**Affiliations:** Surgeon.


### FUNGUS HÆMATODES OF THE BRAIN.

*Case of Fungus Hæmatodes of the Brain, with long-continued Constipation of the Bowels.* By JAS. LAIDLAW, Esq., Surgeon.

SAMUEL KIDMAN, fifty-eight years of age, by occupation a servant. His wife states that it was about two years ago he first complained of a severe fixed pain in the head, immediately over the left temple, which was attended with dimness of sight: these symptoms were relieved, for the time, by the abstraction of blood, by leeches, and by the application of blisters to the part. The attacks, however, became more frequent and of longer duration; the severity of the pain being greater, but still confined to the same spot. Some months after his first attack, he was observed one evening, while at tea, to hang down his head, with a stupid, vacant appearance of countenance, and, upon being spoken to, he was found to be nearly insensible: he was totally unconscious of the presence of his friends, and was with some difficulty (by shaking him and pulling him about,) made aware of it. After that time, he frequently fell into the same comatose state, sometimes as often as eight or nine times in the course of a day; and the attacks of headach, giddiness, &c. returned almost daily. Still he had no paralysis, and, with the exception of a slight loss of appetite, was in tolerable general health, and attended to his domestic duties.

Thus he continued up to the beginning of last April, when one evening, while at tea, he was seized with a fit, which continued about twenty minutes, after which he became calm and tranquil as usual. He was not seen by his medical attendant during the continuance of the fit, but it appears, from the description of his friends, to have been of an epileptic rather than an apoplectic character. Leeches, and a blister, were afterwards applied to the head.

After a lapse of nine days, he had another fit of the same nature, which lasted about the same time, and from which he recovered as from the former. A fortnight after the occurrence of the second fit, he was seized with paralysis of the right side of the body, and with total loss of vision and the power of articulating: this occurred while he was shaving himself. The remedies which had been usually employed were again resorted to, but without any benefit; and he continued in this unhappy condition, suffering little, and often remaining for a day or two in a state of apparent insensibility, until the 11th of July, when he suddenly recovered the power of speech, and from his conversation it was evident his mental powers were unimpaired; but he was very weak, and was evidently sinking. On the 19th of July, he died. For several weeks previous to his death, he had taken very little nourishment, and for *nearly nine weeks* had had no evacuation from the bowels.

*Sectio cadaveris.* Upon opening the head, the membranes on the surface of the brain were found in a perfectly healthy state, with the exception of a slight thickening and opacity of the arachnoid. The right hemisphere of the brain was quite sound and healthy; but, upon cutting into the left, the middle lobe of it was found converted into a mass of fungoid disease: it was considerably larger than natural, and, by pressing upon the petrous portion of the temporal bone, that bone had become partially absorbed, so as to expose the cavities of the ear. The arachnoid membrane, at the inferior part of the tumor, was so much thickened as to form a sort of sac holding the tumor together. No vestige of the left lateral ventricle could be discovered; nor was there any appearance indicating which had been the cineritious and which the medullary part of the brain. Upon removing the diseased mass, it seemed to melt away from the touch, and had a good deal the appearance of the tumor formed by a hernia cerebri. The arteries and nerves at the base of the brain were healthy. In the cavity of the abdomen, the large intestines were found choked up by the accumulations of hardened fæces. The liver was larger, and of a darker colour, than natural; the gallbladder was very much distended, and, upon cutting into it, was found to contain thirty-three gallstones, of the size of peas, and about three ounces of bile. The other viscera were healthy.

One of the most remarkable circumstances in this case was the extreme torpidity of the bowels, so that, for nearly nine weeks previous to his death, no evacuation could be procured. This must have arisen from their impaired nervous sensibility, in consequence of the disease of the brain, as there was no morbid appearance of the bowels themselves. There are two cases published by Dr. Baillie, in which no evacuation could be procured from the bowels, in the one for a period of fifteen weeks, and in the other twenty days; but the obstruction in both these cases was mechanically produced by stricture in the rectum.

*Seymour street, West.*

